# The oilseed rape developmental expression resource: a resource for the investigation of gene expression dynamics during the floral transition in oilseed rape

**DOI:** 10.1186/s12870-020-02509-x

**Published:** 2020-07-21

**Authors:** D. Marc Jones, Tjelvar S. G. Olson, Nick Pullen, Rachel Wells, Judith A. Irwin, Richard J. Morris

**Affiliations:** 1 Crop Genetics, John Innes Centre, Norwich Research Park, Norwich, NR4 7UH UK; 2 Computational and Systems Biology, John Innes Centre, Norwich Research Park, Norwich, NR4 7UH UK

**Keywords:** *Brassica napus*, Oilseed rape, Transcriptomics, Time series, Flowering time, Floral transition, Subfunctionalisation, Neofunctionalisation, Database

## Abstract

**Background:**

Transcriptome time series can be used to track the expression of genes during development, allowing the timing, intensity, and dynamics of genetic programmes to be determined. Furthermore, time series analysis can reveal causal relationships between genes, leading to an understanding of how the regulatory networks are rewired during development. Due to its impact on yield, a developmental transition of agricultural interest in crops is the switch from vegetative to floral growth. We previously reported the collection of genome-wide gene expression data during the floral transition in the allopolyploid crop *Brassica napus* (oilseed rape, OSR). To provide the OSR research community with easy access to this dataset, we have developed the Oilseed Rape Developmental Expression Resource (ORDER; http://order.jic.ac.uk).

**Results:**

ORDER enables users to search for genes of interest and plot expression patterns during the floral transition in both a winter and a spring variety of OSR. We illustrate the utility of ORDER using two case studies: the first investigating the interaction between transcription factors, the second comparing genes that mediate the vernalisation response between OSR and radish (*Raphanus sativus* L.). All the data is downloadable and the generic website platform underlying ORDER, called AionPlot, is made freely and openly available to facilitate the dissemination of other time series datasets.

**Conclusions:**

ORDER provides the OSR research community with access to a dataset focused on a period of OSR development important for yield. AionPlot, the platform on which ORDER is built, will allow researchers from all fields to share similar time series datasets.

## Background

*Brassica napus* (oilseed rape; OSR) is an allopolyploid crop which represents the second largest global source of seed oil after soybean [1]. The progression from vegetative to floral growth is an agriculturally important developmental transition in OSR. The genes involved in this transition in the model plant species *Arabidopsis thaliana* (Arabidopsis) are well characterised [2]. Although Arabidopsis is in the same taxonomic family (Brassicaceae) as OSR, whole genome duplications have occurred in the Brassica lineage [3–5], resulting in multiple OSR orthologues of Arabidopsis genes, many of which have been retained. Determining whether, and how, the OSR orthologues of Arabidopsis flowering time genes have functionally diverged is necessary to translate knowledge from Arabidopsis to OSR.

In previous work we showed that changes in expression dynamics, particularly among genes associated with flowering, are a common evolutionary route for duplicated genes in OSR [6]. This inferred regulatory divergence was assessed by RNA-Seq from tissue samples during the floral transition from both the apex and the leaf, giving rise to a comprehensive OSR transcriptome time series. Providing easy access for researchers in the Brassica community to explore and mine this dataset is the motivation for the development and description of the presented resource.

Repositories exist for expression data [7–9] but ease of access is frequently limited. To address this, query and analysis tools have been made available that can visualise many different experiments and experimental designs [10–13]. These tools facilitate meta-analysis of many disparate datasets, although consequently, the visualisations are often simplified. Other projects are narrower in their scope, providing a frontend to a single dataset. The “Electronic Fluorescent Pictograph” browser displays microarray data from a variety of Arabidopsis organs at many developmental stages [14] as a pictorial heatmap [15]. This provides an intuitive method of interrogating this large dataset, albeit at the cost of flexibility in terms of the types of dataset that can be visualised in this way. The Brassica database, BRAD, is a repository of genetic data for Brassica crops [16], while synteny and gene homology data for many plant species is available as part of the Ensembl Plants database [17]. Trait and genotype data can be submitted to the Brassica Information Portal, facilitating programmatic access to these data and enabling meta-analyses to be conducted [18]. While these centralised repositories exist for Brassica crops, we are not aware of a resource or service that is currently suitable for the visualisation of time series gene expression data.

To query and visualise transcriptome time series datasets in an intuitive manner, we developed The Oilseed Rape Developmental Expression Resource (ORDER; http://order.jic.ac.uk). The database currently provides access to the dataset we have previously reported [6] and can readily be extended to similar datasets. Querying the database using gene identifiers from Arabidopsis and Brassica species finds all OSR genes exhibiting sequence similarity, allowing the expression of homologues to be compared. A sequence-based search function is also available, allowing the database to be queried using genes from species for which homology information is not available within ORDER.

To demonstrate the utility of the website, two case studies are discussed. The first uses adaptive plotting functions to compare the expression of OSR homologues of *AGAMOUS-LIKE 24* (*AGL24*) and *APETALA1* (*AP1*), identifying expression traces consistent with an antagonistic regulatory relationship between genes. The second uses the sequence similarity-based search function to compare the behaviour of *FLOWERING LOCUS C* (*FLC*) orthologues in radish (*Raphanus sativus* L.) to those in OSR. The platform on which ORDER is based, Aionplot, is freely available for download. AionPlot accepts arbitrary time series data, allowing researchers to set up resources like ORDER so users can access and plot datasets.

### Implementation

The website makes use of the Bootstrap framework for the user interface. The Bootstrap framework provides a clean, clear interface that is suitable for different devices. As a result, ORDER is equally usable on computers and tablets. The responsive elements of the website utilise Javascript with jQuery, with the plotting functions using the D3.js library. The database used is MongoDB (version 4.0.6) with the server code written in Python (version 2.7.5), making use of the Flask web development framework. AionPlot is made available as a Docker container. This allows for instances accessible on a local machine, as well as production instances made available to the public. The source code for AionPlot (https://github.com/marc-jones/aionplot) and the data files used to initialise ORDER (https://bitbucket.org/marc-jones/order-data-files) are available in publicly available repositories.

## Results

### OSR developmental time series and ORDER interface

The transcriptome time series we used to study gene expression during development in OSR [6] represents a valuable data resource for the Brassica research community. The dataset contains genome-wide gene expression data from apex and leaf tissue collected before, during, and after the floral transition. Whilst our previous work reported only on a spring variety of OSR, we also collected material from a European winter variety, to allow the vernalisation response to be studied. We present ORDER as a resource to allow users to quickly and easily search this dataset to study the expression dynamics of their genes of interest.

**Fig. 1:**
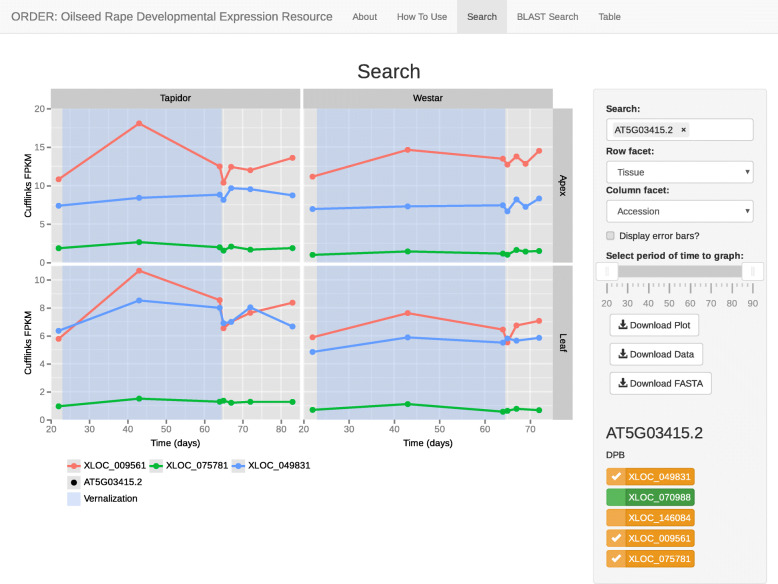
Screenshot of the Search page. The search page allows for gene identifiers and names to be used to search the transcriptome time series dataset. If a search term is for a homologous gene, OSR genes that share sequence conservation to that gene are displayed in the bar on the right. Selecting a particular OSR gene plots the expression profile in all tissues and varieties. The plot settings can be adjusted using the control panel on the right of the screen

**Fig. 2:**
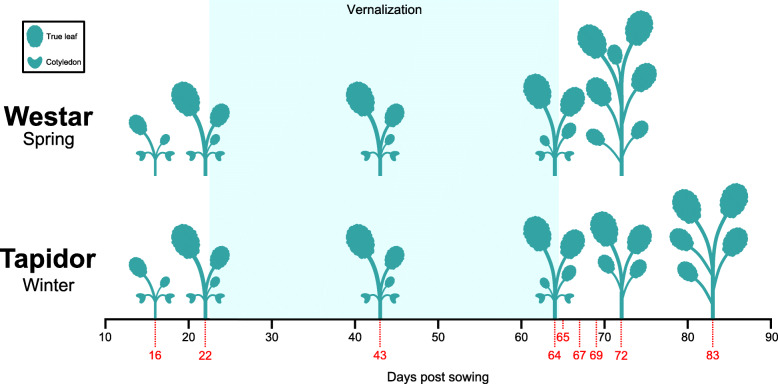
Screenshot of the BLAST Search page. Inserting a nucleotide sequence into the search box prompts the server to perform a search for OSR genes that exhibit sequence similarity. The result of the search is displayed on the sequence search page, and the identified OSR genes are displayed on the Search page to allow users to plot the relevant expression profiles

The dataset can be searched using either sequence similarity to Arabidopsis or Brassica genes (Fig. 1), or sequence similarity to a user submitted sequence (Fig. 2). The OSR gene selected, or OSR genes exhibiting sequence similarity to the selected homologous gene, are displayed below the search box as a checklist (Fig. 1). Selecting one of these OSR genes will plot the corresponding expression profile. Additionally, hovering over each gene in the checklist displays the OSR chromosome the gene is located on. The two facets of the data can be manipulated by the user to facilitate comparisons between the two varieties and two tissues. Plotting expression traces for many homologues simultaneously on the graph can reduce the clarity of the plot. To mitigate this, the drawing of error bars can be toggled, and hovering over gene names in the plot legend highlights the expression trace of that gene in the graph. The interval of time plotted can be controlled with the slider located under the search box, to generate plots focused on a defined period of development. Finally, the generated plot image, the cDNA sequences of the selected genes, and a table of expression values can all be downloaded from this page.

ORDER currently contains homology mappings for published Arabidopsis [TAIR10 [19]], *Brassica oleracea* [Release 43 [17]], *Brassica rapa* [BRAD v3.0 [16, 20]], and OSR [Pantranscriptome [21]] gene models. To allow users to search based on homology to different gene models, ORDER contains a search tool that uses the BLAST algorithm [22] to identify OSR genes with sequences similar to a user submitted sequence (Fig. 2). To plot expression patterns of the genes that are identified by the algorithm, the user returns to the Search page and selects the checkboxes corresponding to the identified genes.

**Fig. 3:**
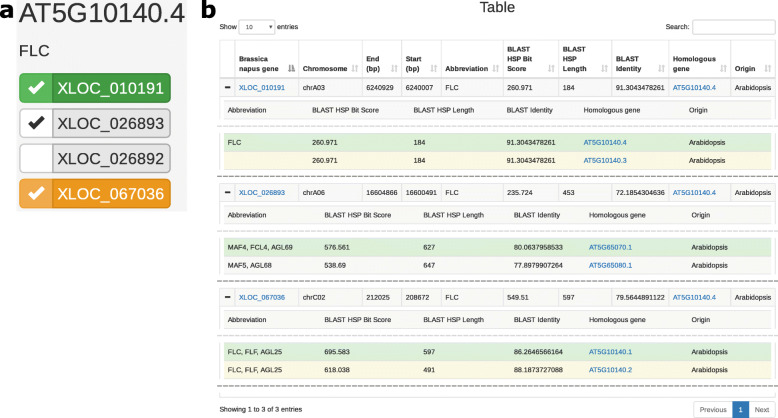
Screenshot of the Table page. Selecting OSR genes on the Search page (**a**) creates a row in the table (**b**). Displayed on each row is the OSR gene name, the chromosome and chromosome position where the gene is located, and if applicable, details about the degree of sequence conservation between the homologous gene searched for and the OSR gene selected. Additional sequence similarity information can be accessed by clicking the + symbol on the left of the table. Due to the many-to-many mapping of OSR genes to homologous genes, a colour code is used. In this case, the user has searched for OSR genes exhibiting homology to the Arabidopsis gene *FLC*, and specifically, to the splice isoform AT5G10140.4. The OSR gene XLOC_010191 shows highest sequence conservation to AT5G10140.4, and is coloured green. XLOC_026893, however, shows greatest sequence similarity to the Arabidopsis gene *MADS AFFECTING FLOWERING 4* (*MAF4*), rather than *FLC*, and is coloured white. Genes that are coloured yellow display greatest similarity to the gene searched for, although to a different splice isoform than the one the user searched for. In the case of XLOC_067036, it is most similar to the *FLC* splice isoform AT5G10140.1. Dashed lines in (**b**) indicate that table rows have been omitted for clarity

To compare the results identified using ORDER and previous publications, it is useful to determine where in the genome the genes are located. To facilitate this, ORDER generates an information table for the genes that are selected on the Search page (Fig. 3). This table contains the chromosome on which the genes are located, as well as their start and end positions on that chromosome. If applicable, the gene name used to query the database is displayed, along with the percentage sequence identity, sequence similarity score, and length of the sequence identified by the BLAST algorithm as being similar between the query gene or sequence. In addition, other genes identified as having sequence similarity to the selected OSR gene by the BLAST algorithm can be viewed. The colour of the rows in the sub-tables correspond to the best matching gene models within each set of gene models. If the OSR gene is the best match identified, or is a splice isoform of the best match, then the row will be coloured green or orange respectively. This colouration is also used on the Search page, to help determine the genes most likely to be homologues of the gene entered in the search box. Links to other community resources are integrated on this page. The OSR gene name is a hyperlink that takes the user to the position of the gene in a genome browser of the OSR genome [23], while the Arabidopsis and *B. oleracea* gene name hyperlinks take the user to the corresponding entry on The Arabidopsis Information Resource or Ensembl Plants respectively [17, 24]. In the following sections we demonstrate, through two case studies, the utility of ORDER for investigating aspects of flowering time regulation in OSR.

### Case study 1: potential regulatory interactions between floral integrators

*AGL24* is a known Arabidopsis floral integrator that is expressed in the vegetative meristem and promotes the floral transition [25, 26]. Mutants lacking *AGL24* exhibit delayed flowering, while lines overexpressing the gene are early flowering [25, 26]. Plants overexpressing *AGL24* also display a partial reversion of floral meristems into inflorescence shoots, suggesting that the gene helps to maintain the meristem in an inflorescent state [27]. Thus, although the gene initially promotes the floral transition, expression of the gene has to be downregulated as the flower develops to prevent floral reversion [27]. This repression is mediated directly by a second Arabidopsis floral integrator, *AP1* [27–29].

**Fig. 4:**
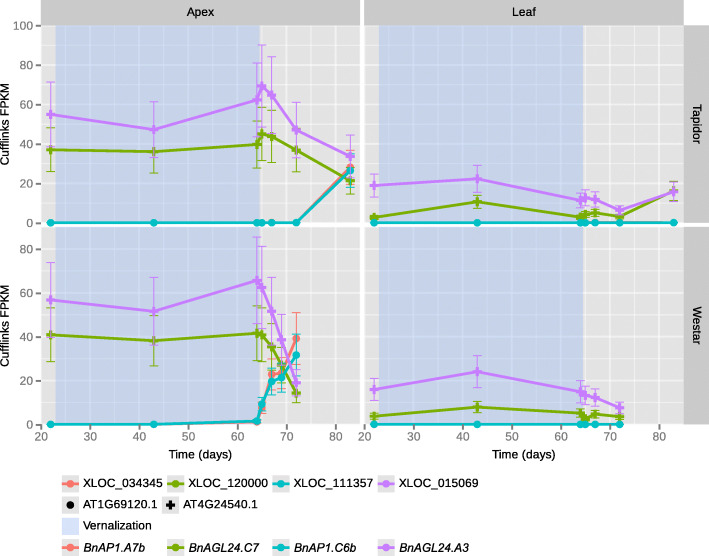
Expression profiles of *BnAGL24* and *BnAP1* genes reveals potential repression. The expression values and the 95% confidence intervals of those expression values as computed by Cufflinks are displayed. The expression profiles of OSR homologues of *ALG24* (AT4G24540.1) and *AP1* (AT1G69120.1) are plotted. In this figure, the tissue and variety divisions have been swapped relative to Fig. 1 using the plotting controls. Plotting the figure in this manner allows for the timing of the expression changes to be more easily compared between varieties. In the apex, the expression of *BnAGL24* genes (XLOC_015069 and XLOC_120000) decreases after the cold treatment, with the expression of *BnAP1* genes (XLOC_034345 and XLOC_111357) increasing. The bottom line of the legend has been added for consistency with the main text, and is not present on the visualisations generated by ORDER

To investigate the behaviour of *AP1* and *AGL24* in OSR, ORDER was used to plot the expression profiles of OSR homologues (*BnAP1*, *BnAGL24*). Within ORDER, eight gene models show homology to *AP1* (Supplementary Table 1). Four copies of *BnAP1* are similarly upregulated during the floral transition in the apex in both varieties (*BnAP1.A7b*, *BnAP1.C6b* [Fig. 4], *BnAP1.A7a, BnAP1.C6a* [Supplementary figure 1]), while *BnAP1.A2.Random* is upregulated in the apex to a much higher degree in Tapidor relative to Westar (Supplementary figure 1b). Comparing the copies of *BnAP1* that are upregulated between varieties, a difference in timing is observed, with the expression increase beginning on day 65 of the time series for Westar and between day 72 and day 83 of the time series for Tapidor. This difference aligns well with the offset in developmental timing observed between the varieties (Supplementary figure 2). The remaining three copies are much more lowly expressed in comparison and show a slight expression response to vernalisation treatment (Supplementary figure 3). Four *BnAGL24* gene models are identified, on chromosomes A1, A3, C1, and C7 (Supplementary Table 1). The expression profiles of the four genes can be categorised into two behaviours. *BnAGL24.A3* and *BnAGL24.C7* are both highly expressed before the vernalisation treatment, and remain at similar expression levels throughout the treatment (Fig. 4). After the treatment both copies decrease to similar levels, although the rate of decrease is slower in Tapidor relative to Westar (Fig. 4). This pattern of expression closely resembles the expression of *AGL24* in Arabidopsis [27]. The other two *BnAGL24* genes, *BnAGL24.A1* and *BnAGL24.C1*, show markedly different behaviour, increasing slightly during the vernalisation treatment, then rapidly increasing in expression post-treatment, followed by a decrease towards the end of the time series (Supplementary figure 4).

In order to compare the expression profiles of *BnAP1* and *BnAGL24*, homologues of the two genes were visualised together with the ORDER plotting feature. For clarity, the two most highly expressed *BnAP1* genes (*BnAP1.A7b* and *BnAP1.C6b*) and the two *BnAGL24* genes expressed most similarly to Arabidopsis (*BnAGL24.A3* and *BnAGL24.C7*) were plotted (Fig. 4). The post-vernalisation expression level of *BnAP1.A7b* and *BnAP1.C6b* increases concurrently with the decrease in expression of *BnAGL24.A3* and *BnAGL24.C7* in the apex of both varieties (Fig. 4). These expression profiles are consistent with the repression of *BnAGL24* homologues by *BnAP1*, as findings from Arabidopsis would suggest [27–29]. That the expression level of the *BnAGL24* genes begins to decrease before *BnAP1* genes begin to increase in Tapidor suggests that other proteins may also be playing a role in the repression of *BnAGL24* in OSR.

### Case study 2: comparison of *FLOWERING LOCUS C* (*FLC*) orthologues from radish

Radish (*Raphanus sativus* L.) is a diploid plant grown as a root crop and, like OSR, is also a member of the taxonomic family Brassicaceae [30]. Arabidopsis genes are expected to have multiple homologues in the radish genome, as the genome triplication event present in the evolutionary history of the Brassica lineage [3–5] is also shared with radish [30]. Several lines of evidence suggest that aspects of the flowering time pathway are conserved between radish and Brassica species [31]. In particular, three radish *FLC* orthologues (*RsFLC1*, *RsFLC2*, and *RsFLC3*) have been identified, all of which repress flowering when overexpressed in Arabidopsis [32]. Expression of the *RsFLC1* gene was also found to be higher in a late-bolting radish variety relative to an early-bolting variety, consistent with the role *FLC* plays in Arabidopsis and Brassica species [33].

In order to compare the *RsFLC* genes with those of OSR, the gene sequences for *RsFLC1*, *RsFLC2*, and *RsFLC3* were used to query ORDER using the BLAST search feature. 18, 22, and 20 BLAST hits were identified within the ORDER database for *RsFLC1*, *RsFLC2*, and *RsFLC3* respectively (Supplementary Table 2). In addition to *BnFLC*, OSR orthologues of the Arabidopsis genes *MADS AFFECTING FLOWERING 1–4* and *AGL6* were identified by the BLAST algorithm. However, the highest scoring BLAST hits for *RsFLC1*, *RsFLC2*, and *RsFLC3* were all *BnFLC* genes, and these genes are therefore regarded as the corresponding orthologous OSR genes (Table 1).

**Table 1:** Details of the radish *FLC* orthologues identified in OSR

Radish gene	OSR gene	Chromosome	End (bp)	Start (bp)	BLAST HSP bit score	BLAST HSP length (bp)	BLAST identity (%)
Cultivar WK10039 FLC1 (KP027017)	XLOC_047868	chrA10	15,003,589	14,998,200	434.10	381	85.83
Cultivar WK10039 FLC2 (KP027026)	XLOC_006960	chrA02	138,121	134,159	342.12	274	85.77
Cultivar WK10039 FLC3 (KP027035)	XLOC_012674	chrA03	1,364,314	1,360,824	412.45	332	88.55

The GenBank identifier for the radish gene sequence used is given in parentheses in the ‘Radish gene’ column. The OSR gene that had the highest high-scoring segment pair (HSP) score, as calculated by the BLAST algorithm, is reported here. The data in this table is copied directly from the ORDER table view.

**Fig. 5:**
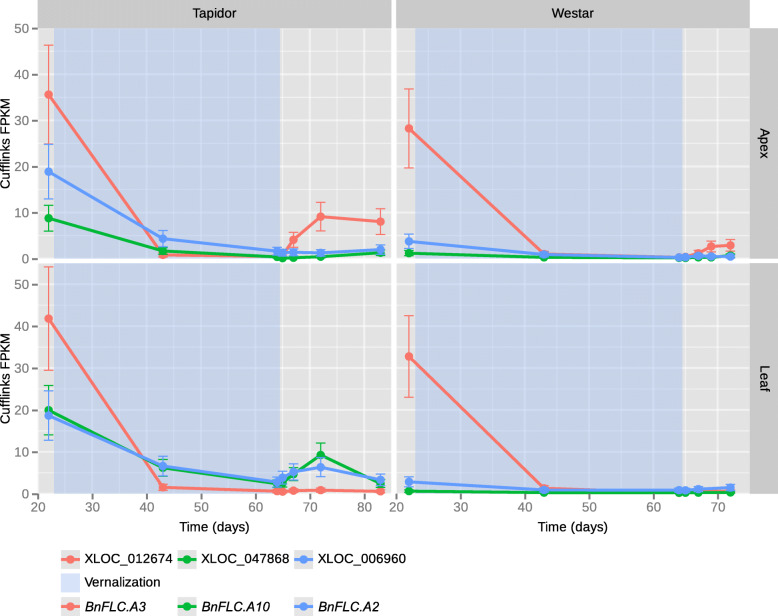
Expression profiles of the closest OSR homologues of the radish *FLC* genes. The expression values and the 95% confidence intervals of those expression values as computed by Cufflinks are displayed. The relationships between the radish and OSR gene names are as follows: *RsFLC1* (XLOC_012674; *BnFLC.A3*), *RsFLC2* (XLOC_006960; *BnFLC.A2*), and *RsFLC3* (XLOC_047868; *BnFLC.A10*). The bottom line of the legend has been added for consistency with the main text, and is not present on the visualisations generated by ORDER

All identified OSR orthologues were located on the A genome, consistent with the closer evolutionary relationship between radish and *B. rapa* [30]. Interestingly, the three orthologous *BnFLC* genes appear to have diverged in their expression dynamics. Two of the three genes (*BnFLC.A2* and *BnFLC.A10*) are more highly expressed before the cold treatment in both tissues in Tapidor compared to Westar (Fig. 5). During the cold treatment, expression of all three OSR *FLC* genes decreases, and remains lower than the starting levels after cold. This is consistent with *BnFLC* genes being epigenetically silenced as a result of cold exposure, as is the case in Arabidopsis [34]. As Westar is a spring variety, and therefore does not require vernalisation to flower, the high expression of *BnFLC.A3* before the cold treatment (at a level similar to that observed in Tapidor) suggests that repression of this *FLC* gene is not required for flowering. The behaviour of *BnFLC.A10* is consistent with another study, that found the gene to be differentially expressed between winter and spring OSR varieties [35]. As is observed for some Arabidopsis *FLC* alleles [34], *BnFLC* genes sometimes exhibit reactivation after cold. *BnFLC.A3* expression partially recovers after the vernalisation treatment in the apex in both varieties, while a degree of reactivation is also observed for *BnFLC.A2* and *BnFLC.A10* in leaf tissue in Tapidor (Fig. 5). This may be due to the length of cold, or the severity of cold, not being sufficient to lead to complete epigenetic silencing of these genes. Alternatively, it may be due to the silencing of these *BnFLC* genes being transient, as is the case for *FLC* orthologues in perennial plants [36].

## Discussion

Since the advent of microarrays and RNA sequencing, many studies have collected gene expression data on a genome-wide scale across time. Although the data from these studies are deposited in online, public repositories, very few can be queried without a minimum amount of bioinformatics first being conducted to quantify gene expression levels. The objective of ORDER is to create a Brassica community resource to allow users to easily search for OSR genes of interest and plot expression profiles. ORDER provides access to developmental time series from two OSR varieties [6] and can be readily extended to include further datasets. To demonstrate the utility of ORDER for exploring the transcriptomic time series, two examples of using the website were outlined. Future developments to the website will be guided by user feedback and could for instance focus on further integration with other Brassica crop resources and improved tools for data analysis.

In addition to the community need for a gene expression visualisation tool for Brassica species, methods to disseminate time series data in an intuitive manner are lacking. ORDER is presented as a resource for the Brassica community, however, the underlying platform, AionPlot, is agnostic towards the time series data used to initialise the website. AionPlot therefore allows websites to be created for other time series datasets with ease.

## Conclusions

The dual search functions allow full access to the dataset, allowing users to search using sequence similarity to Arabidopsis genes or to user submitted sequences. The organisation of the website is such that any time series data, regardless of origin, can be visualised using the website code underlying ORDER. That website code is provided to the community as the tool AionPlot.

### Availability and requirements

Project name: ORDER: Oilseed Rape Developmental Expression Resource. Project home page: order.jic.ac.uk. Operating system(s): Platform independent. Programming language: Python and Javascript. Other requirements: None. License: GNU GPL. Any restrictions to use by non-academics: Compliance with GNU GPL license.

## Materials and methods

### Plant growth, sample preparation, and gene expression quantification

*Brassica napus* cv. Westar and *Brassica napus* cv. Tapidor plants were grown as per the method described in Jones et al. (2018). Both varieties were sampled between BBCH stages 13 and 51 [37]. For Westar, the first true leaf and shoot apex of each plant were sampled at 22, 43, 64, 65, 67, 69 and 72 days after sowing. Tapidor tissue was sampled at the same times except at day 69 after sowing, with an additional time point taken 83 days after sowing, to ensure that the same developmental period (BBCH stages 13 through 51) were represented for both varieties. Samples were prepared and gene expression levels were quantified using the methods previously published [6].

## Supplementary information

**Additional file 1: Figure S1.** Expression profiles of additional *BnAP1* genes that increase in expression after the cold treatment. The expression values and the 95% confidence intervals of those expression values as computed by Cufflinks are displayed. In addition to the two *BnAP1* genes that exhibit an increase in expression after the cold treatment in Fig. 4 in the main text (XLOC_034345 and XLOC_111357), this plot displays the expression profiles of the other three *BnAP1* genes that display that behaviour. The bottom line of the legend has been added for consistency with the main text, and is not present on the visualisations generated by ORDER. a Expression profiles from both tissues are displayed. b Expression profiles from the apex only are displayed.

**Additional file 2: Figure S2.** Cartoon of the developmental stages of Tapidor and Westar at each time point. Plant tissue was sampled on the days indicated by red dotted lines and numbers. The plant silhouettes represent the approximate number of full leaves at the indicated points in development, allowing the developmental stage of the plants to be estimated.

**Additional file 3: Figure S3.** Expression profiles of lowly expressed *BnAP1* genes. The expression values and the 95% confidence intervals of those expression values as computed by Cufflinks are displayed. The bottom line of the legend has been added for consistency with the main text, and is not present on the visualisations generated by ORDER.

**Additional file 4: Figure S4.** Expression profiles of additional *BnAGL24* genes. The expression values and the 95% confidence intervals of those expression values as computed by Cufflinks are displayed. In addition to the two *BnAGL24* genes that exhibit a decrease in expression after the cold treatment in Fig. 4 in the main text (XLOC_015069 and XLOC_120000), this plot displays the expression profiles of the other two *BnAGL24* genes. The bottom line of the legend has been added for consistency with the main text, and is not present on the visualisations generated by ORDER.

**Additional file 5: Table S1.** Genomic location and homology information for all *BnAP1* and *BnAGL24* genes in the ORDER database. Except for the “*Brassica napus* gene name” column, this table represents the data available from the ORDER table view.

**Additional file 6: Table S2.** Genomic location and homology information for all BLAST hits to *RsFLC1*, *RsFLC2*, and *RsFLC3* identified in the ORDER database.

## Data Availability

The ORDER website may be accessed at the web address: http://order.jic.ac.uk. The transcriptomic data presented in ORDER is available in a format compatible with AionPlot from a Bitbucket repository (https://bitbucket.org/marc-jones/order-data-files). The raw reads have been deposited in the NCBI Sequence Read Archive under the BioProject numbers PRJNA398789 and PRJNA565743. All code for AionPlot is available from GitHub (https://github.com/marc-jones/aionplot) while Docker images are maintained in DockerHub (https://hub.docker.com/r/dmarcjones/aionplot-flaskapp).

## Abbreviations

ORDER: Oilseed Rape Developmental Expression Resource
OSR: Oilseed Rape, *Brassica napus*
AGL24: *AGAMOUS-LIKE 24*
AP1: *APETALA1*
FLC: *FLOWERING LOCUS C*
