# Supplementary figures and images for: The oilseed rape developmental expression resource: a resource for the investigation of gene expression dynamics during the floral transition in oilseed rape
Source: BMC Plant Biol. 2020 Jul 21;20:344. doi: 10.1186/s12870-020-02509-x (PMC7374918; doi:10.1186/s12870-020-02509-x)

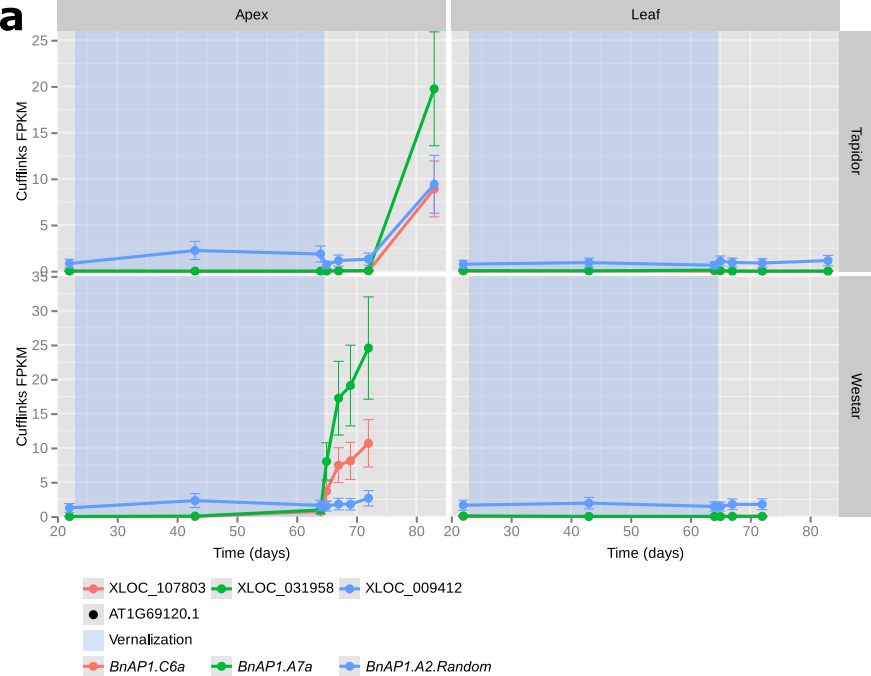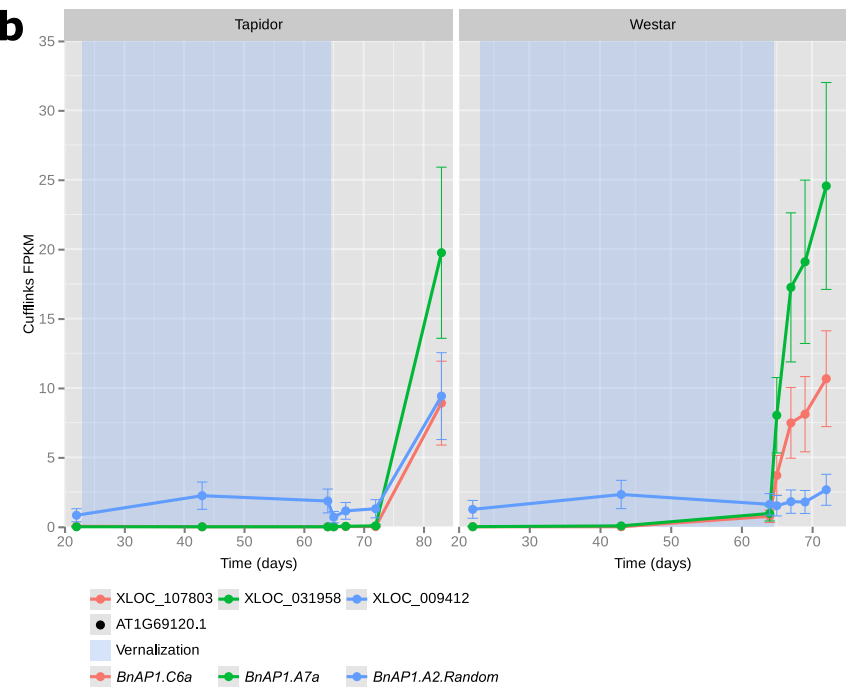

Supplement: Supplementary file 1 — Additional file 1: Figure S1. Expression profiles of additional BnAP1 genes that increase in expression after the cold treatment. The expression values and the 95% confidence intervals of those expression values as computed by Cufflinks are displayed. In addition to the two BnAP1 genes that exhibit an increase in expression after the cold treatment in Fig. 4 in the main text (XLOC_034345 and XLOC_111357), this plot displays the expression profiles of the other three BnAP1 genes that display that behaviour. The bottom line of the legend has been added for consistency with the main text, and is not present on the visualisations generated by ORDER. a Expression profiles from both tissues are displayed. b Expression profiles from the apex only are displayed. [file 12870_2020_2509_MOESM1_ESM.pdf]

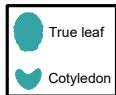

**Westar**  
Spring

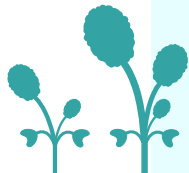

Vernalization

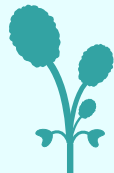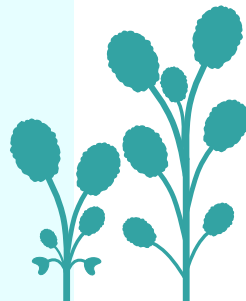

**Tapidor**  
Winter

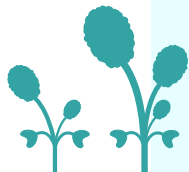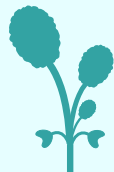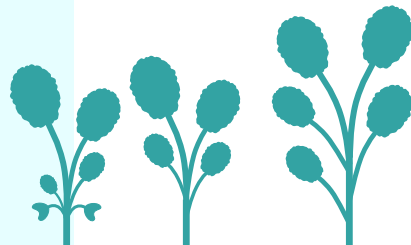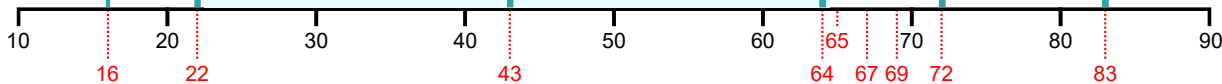

Days post sowing

Supplement: Supplementary file 2 — Additional file 2: Figure S2. Cartoon of the developmental stages of Tapidor and Westar at each time point. Plant tissue was sampled on the days indicated by red dotted lines and numbers. The plant silhouettes represent the approximate number of full leaves at the indicated points in development, allowing the developmental stage of the plants to be estimated. [file 12870_2020_2509_MOESM2_ESM.pdf]

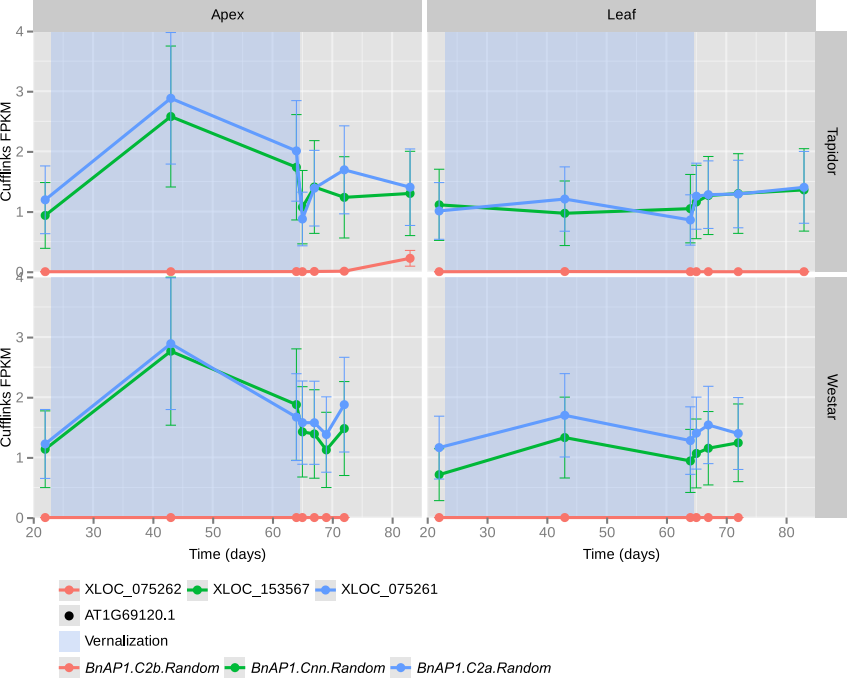

Supplement: Supplementary file 3 — Additional file 3: Figure S3. Expression profiles of lowly expressed BnAP1 genes. The expression values and the 95% confidence intervals of those expression values as computed by Cufflinks are displayed. The bottom line of the legend has been added for consistency with the main text, and is not present on the visualisations generated by ORDER. [file 12870_2020_2509_MOESM3_ESM.pdf]

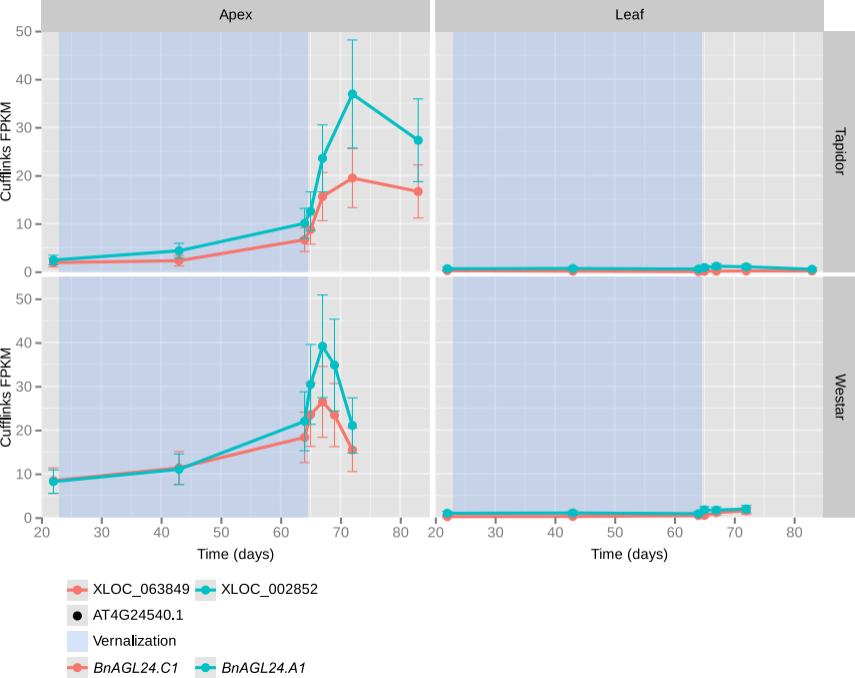

Supplement: Supplementary file 4 — Additional file 4: Figure S4. Expression profiles of additional BnAGL24 genes. The expression values and the 95% confidence intervals of those expression values as computed by Cufflinks are displayed. In addition to the two BnAGL24 genes that exhibit a decrease in expression after the cold treatment in Fig. 4 in the main text (XLOC_015069 and XLOC_120000), this plot displays the expression profiles of the other two BnAGL24 genes. The bottom line of the legend has been added for consistency with the main text, and is not present on the visualisations generated by ORDER. [file 12870_2020_2509_MOESM4_ESM.pdf]
